# Immunohistochemical Correlation of Matrix Metalloproteinase-2 and Tissue Inhibitors of Metalloproteinase-2 in Tobacco Associated Epithelial Dysplasia

**DOI:** 10.1155/2014/197813

**Published:** 2014-01-27

**Authors:** Dipshikha Bajracharya, Bijayatha Shrestha, Asha Kamath, Aparna Menon, Raghu Radhakrishnan

**Affiliations:** ^1^Manipal Education and Medical Group, Manipal University, Manipal 576104, India; ^2^Kasturba Medical College, Manipal University, Manipal 576104, India; ^3^Department of Oral and Maxillofacial Pathology, Manipal College of Dental Sciences, Manipal University, Manipal, Karntaka 576104, India

## Abstract

*Aim*. To study the immunohistochemical expression of matrix metalloproteinase and tissue inhibitors of matrix metalloproteinase-2 in different histological grades of tobacco associated epithelial dysplasia and correlate the association between these proteases. Potentially malignant oral disorders (PMODs) progressing to oral cancer are related to the severity of epithelial dysplasia. *Methods*. A retrospective immunohistochemical study was carried out on 30 clinically and histologically proven cases of leukoplakia with dysplasia and 10 cases of normal buccal mucosa using anti-MMP-2 and anti-TIMP-2 monoclonal antibodies. *Results*. Mann Whitney *U* test, for comparing the expression of both MMP-2 and TIMP-2 in normal mucosa with dysplasia, was highly significant (*P* < 0.001). Kruskal-Wallis test to compare the median score of MMP-2 and TIMP-2 in different grades of dysplasia showed statistical significance (*P* < 0.001), and a Spearman's correlation between MMP-2 and TIMP-2 through different grades of dysplasia and cells observed showed positive correlation. *Conclusion*. Concomitant increase in the expression of both MMP-2 and TIMP-2 suggested that the activation of MMP-2 is dependent on TIMP-2 acting as a cofactor. Changes in TIMP-2 levels are considered important because they directly affect the level of MMP-2 activity.

## 1. Introduction

Oral leukoplakia associated with tobacco is the most common among the potentially malignant oral disorders (PMODs). Oral leukoplakia progressing to oral squamous cell carcinoma (OSCC) has been accepted to be directly related to the severity of epithelial dysplasia [1]. Although the malignant transformation rate for leukoplakia ranges from 0.6% to 18% [2], there are no definitive biological indicators to assess the risk of malignant transformation. These uncertainties in determining the true nature of dysplastic lesions and subjective variation in diagnostic pathology using traditional methods have paved the way for the use of biomarkers.

In recent years the focus has shifted to the host related factors, which play a pivotal role in determining malignant transformation from its potentially malignant state. One of the important host related factors that influences this malignant progression and subsequent invasion belongs to a family of proteins called matrix metalloproteinase (MMP) [3–6]. This zinc dependent enzyme primarily regulates many developmental processes such as branching morphogenesis and angiogenesis [7]. In addition, MMPs contribute in wound healing and extracellular matrix degradation. The activity of MMPs is influenced by enzymes belonging to a family of Serpins, called tissue inhibitors of matrix metalloproteinase (TIMP) [8]. Any imbalance in this regulatory mechanism involving MMP and TIMP may have a telling effect on the net matrix degradation resulting in basement membrane breakdown, tumor invasion, and subsequent spread [9, 10].

MMP-2/72 kD type IV collagenase is the most widely distributed member of MMPs, in which along with MMP-9 cleaves the type IV collagen presents in the basement membrane, which is a prerequisite for tumor cell migration [7, 8]. Activation of pro-MMP-2 consists of various overlapping processes, involving a complex formation that consists of the zymogen MMP-2, MT1-MMP, and TIMP-2. However, the association between MMP-2 and TIMP-2 and their involvement in PMOD is yet to be clarified. Elucidation of the biological activity of these enzymes may determine their correlation in tumor progression.

## 2. Materials and Methods

### 2.1. Case Selection and Inclusion and Exclusion Criteria

A retrospective immunohistochemical analysis was carried out on 30 formalin fixed paraffin embedded (FFPE) specimen, which were clinically and histologically proven cases of leukoplakia with dysplasia. Ten apparently normal oral mucosal tissues, which were age and sex matched with positive tobacco status, obtained at the time of extraction of teeth with patients consent, were used as controls. Prior to carrying out the study, University Ethical Committee approval was obtained. Selection of cases was based on strict inclusion and exclusion criteria. All the cases in the study group were male patients who were in 4th–6th decade of life having a positive history of tobacco habit. This was because most of the reported dysplastic lesions in this region was primarily among elderly men, who develop the lesion following prolonged tobacco use. Only those cases which were present on the buccal mucosa or the alveolar mucosa, with histological confirmation of epithelial dysplasia, were included. Based on the criteria put forth by Lumerman et al. [11], 30 cases of epithelial dysplasia were graded into three categories of mild, moderate, and severe with 10 cases in each group (Table 1).

### 2.2. Antibodies

Immunohistochemistry was carried out by polymer chain two-step indirect technique on each of these cases, using mouse anti-MMP-2 (NCL-MMP2-507 clone 17B11) and anti-TIMP-2 (NCL-TIMP2-487 clone 46E5) monoclonal antibody obtained from Leica Biosystems, Newcastle Ltd. While the tissue sections of inflammatory bowel disease served as positive controls for MMP-2 staining, the placental tissue was used as a positive control for TIMP-2 staining. The endothelial cells were considered as internal positive control for all the cases observed.

### 2.3. Immunohistochemistry

4 *μ*m thick sections on APES coated glass slides were deparaffinized and washed in distilled water for 3 minutes. Antigen retrieval was done using antigen unmasking solution (1 mM of Tris-EDTA buffer, pH 9.0), which was preheated in microwave at 800 W for 5 minutes and heated at 600 W for 10 minutes. The sections were then allowed to cool in the same solution. Endogenous peroxidase blocking was done in 5% hydrogen peroxide for 20 minutes and blocking of nonspecific antibody binding was ensured by incubating the sections in 5% casein in Tris buffered solution (TBS) for 1 hour. Following a brief wash in TBS, the sections were incubated at room temperature in moist chamber for 2 hours with mouse anti-MMP-2 diluted at 1 : 20 and mouse anti-TIMP-2 diluted at 1 : 20. Post primary block was done by polymer penetration enhancer, 0.09% Proclin 950, for 30 minutes. Sections were then incubated at room temperature for 30 minutes with anti-mouse IgG-Poly-HRP. Between each step the sections were washed with 2 changes of TBS for 5 minutes each. The reaction product was visualized with DAB stained for 5 minutes and Mayer's haematoxylin counter stain.

### 2.4. Immunohistochemical Analysis

The expression of the MMP-2 and TIMP-2 was assessed in the basal and parabasal layers of cells of the dysplastic epithelium along with the fibroblasts in the lamina propria. A total of 100 basal and parabasal layers and fibroblasts were examined in each of the 5 different fields at 40x magnification to study the expression pattern. To eliminate any interobserver bias the expression of MMP-2 and TIMP-2 was studied independently by two observers. A semiquantitative analysis was performed based on the proportion of positively stained cells. A positive cell demonstrated a diffuse brown signal in the cytoplasm of cells, independent of its intensity [12]. The immunoreactivity in the basal and parabasal layers of the epithelium and fibroblasts of the lamina propria was scored from 0 to 3 based on the criteria put forth by Roukolainen et al. [13]. The expression was considered to be positive when >1% of either the basal or parabasal cells or fibroblasts showed positive staining. The staining expression was considered weakly positive (+), when 1–25% of the cells were found positive, moderately positive (++), when 26%–50% of cells were positive and strongly positive (+++) when >50% of cells were positive for both MMP-2 and TIMP-2.

### 2.5. Statistical Analysis

SPSS for Windows computer program was used for statistical analysis. Kendall's tau-b test was applied to assess the measure of agreement between two observers. Mann Whitney *U* test was applied for comparing the expression of MMP-2 and TIMP-2 in normal oral mucosa and dysplasia. Kruskal-Wallis test was used to compare the median score of MMP-2 and TIMP-2 across different cells and different grades of dysplasia. Spearman's bivariate correlation was used to analyze the expression of MMP-2 with TIMP-2 in the basal and parabasal layers of cells and fibroblasts. *P* < 0.05 was considered significant for all statistical analyses.

## 3. Results

**Figure 1:**
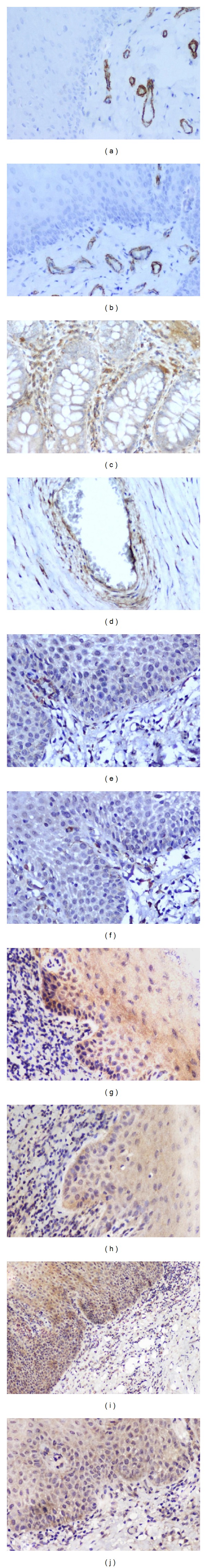
Photomicrographs showing the immunohistochemical expression of MMP-2 and TIMP-2 in tissue sections. (a) Normal buccal mucosa showing negative expression for MMP-2, ×20. (b) Normal buccal mucosa showing negative expression for TIMP-2, ×20. (c) Inflammatory bowel used as positive control for MMP-2, ×20. (d) Placenta used as positive control for TIMP-2, ×20. (e) Mild dysplasia showing weak positive expression for MMP-2, ×20. (f) Mild dysplasia showing weak positive expression for TIMP-2, ×20. (g) Moderate dysplasia showing moderate positive expression of MMP-2, ×20. (h) Moderate dysplasia showing moderate positive expression of TIMP-2, ×20. (i) Severe dysplasia showing strong positive expression of MMP-2, ×20. (j) Severe dysplasia showing strong positive expression of TIMP-2, ×20.

In all the 10 cases of normal buccal mucosa, the expression of MMP-2 (Figure 1(a)) and TIMP-2 (Figure 1(b)) in the basal cells and parabasal cells of the epithelium and in the fibroblasts of lamina propria was negative except in the endothelial cells, which showed positive expression. However, MMP-2 and TIMP-2 when positive in the dysplastic epithelium were comparable to the staining of inflammatory bowel (Figure 1(c)) and placenta (Figure 1(d)), which were used as positive controls, respectively.

The expression score of MMP-2 and TIMP-2 in each of the 10 cases of leukoplakia with mild dysplasia (Figures 1(e) and 1(f)), moderate dysplasia (Figures 1(g) and 1(h)), and severe dysplasia (Figures 1(i) and 1(j)) in the basal and the parabasal cells of the epithelium and in the fibroblasts of the lamina propria is shown in Table 2. Kendall tau-b measure of agreement between the two observers was 0.9 for MMP-2 and 0.93 for TIMP-2. Mann Whitney *U* test, to compare the expression scores of MMP-2 and TIMP-2 of normal oral mucosa with epithelial dysplasia, was statistically significant (*P* < 0.001).

**Figure 2:**
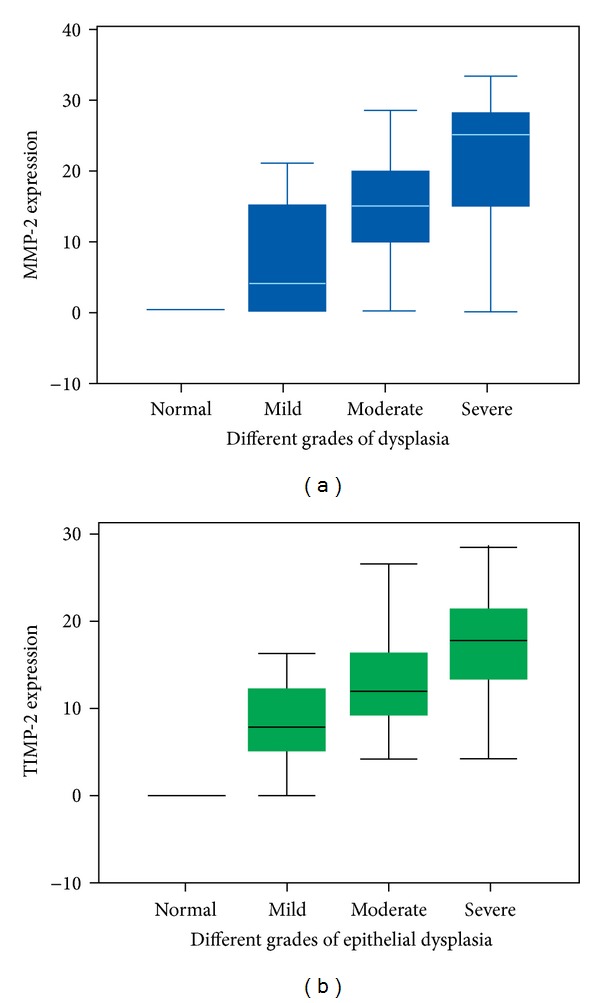
Box plot comparing the median frequency of expression in each the histological grades of dysplasia. (a) MMP-2 expression; (b) TIMP-2 expression.

Descriptive statistics with regard to the median frequencies of cells stained for MMP-2 expression revealed interquartile range of 2 : 4 in mild dysplasia, 2 : 15 in moderate dysplasia, and 2 : 25 in severe dysplasia (Figure 2(a)). Similarly the proportion of cells stained for TIMP-2 expression revealed a median frequency of 2 : 7.50 in mild dysplasia, 2 : 12 in moderate dysplasia, and 2 : 17.50 in severe dysplasia (Figure 2(b)).

**Figure 3:**
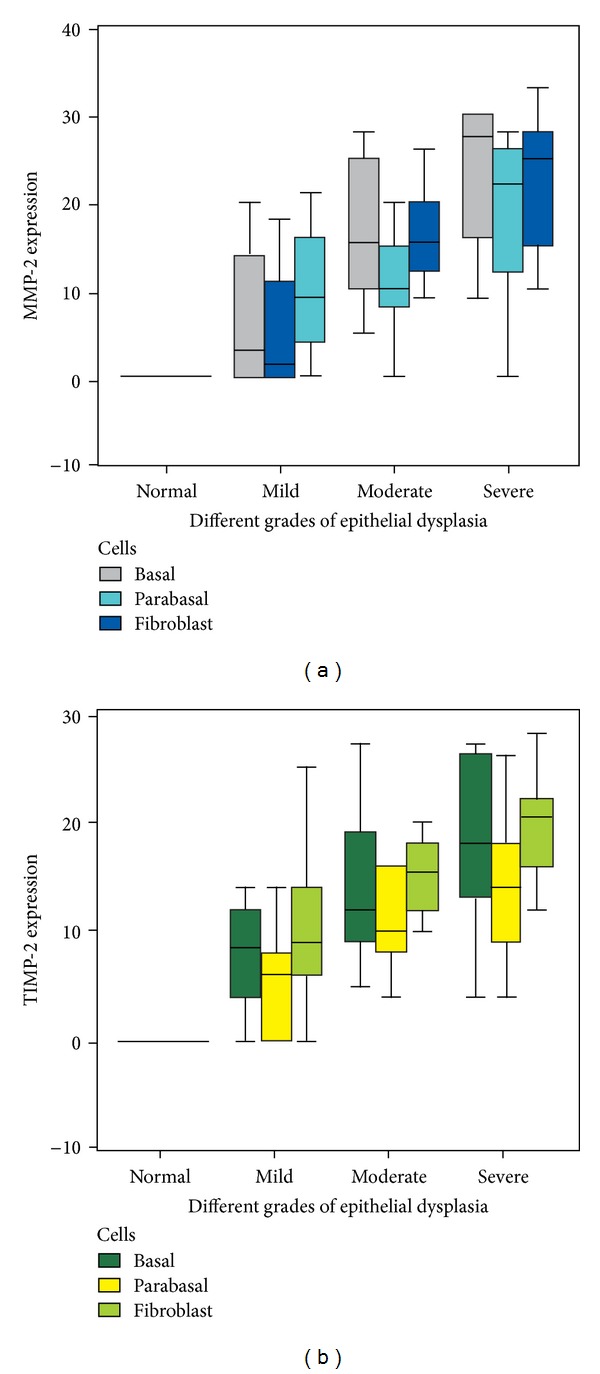
Box plot comparing the median measure of expression across different cells studied in each of the histological grades of dysplasia. (a) MMP-2 expression; (b) TIMP-2 expression.

Cell wise distribution of the median frequencies for MMP-2 showed 2 : 9.50 in basal layer of cells, 2 : 4 in the parabasal cells, and 2 : 12 in fibroblasts of lamina propria (Figure 3(a)). Similarly, the median frequencies of expression for TIMP-2 were 2 : 9.50 in the basal layer of cells, 2 : 7.50 in parabasal cells, and 2 : 12 in the fibroblasts of lamina propria (Figure 3(b)). Kruskal-Wallis test, which used to compare the mean rank of MMP-2 and TIMP-2 across different grades of dysplasia, was significant for each of the cells studied at 1% level of significance (Table 3). However, Kruskal-Wallis test across different cells was statistically not significant with *P* = 0.275 for MMP-2 and *P* = 0.154 for TIMP-2 at 0.1% level of significance.

**Figure 4:**
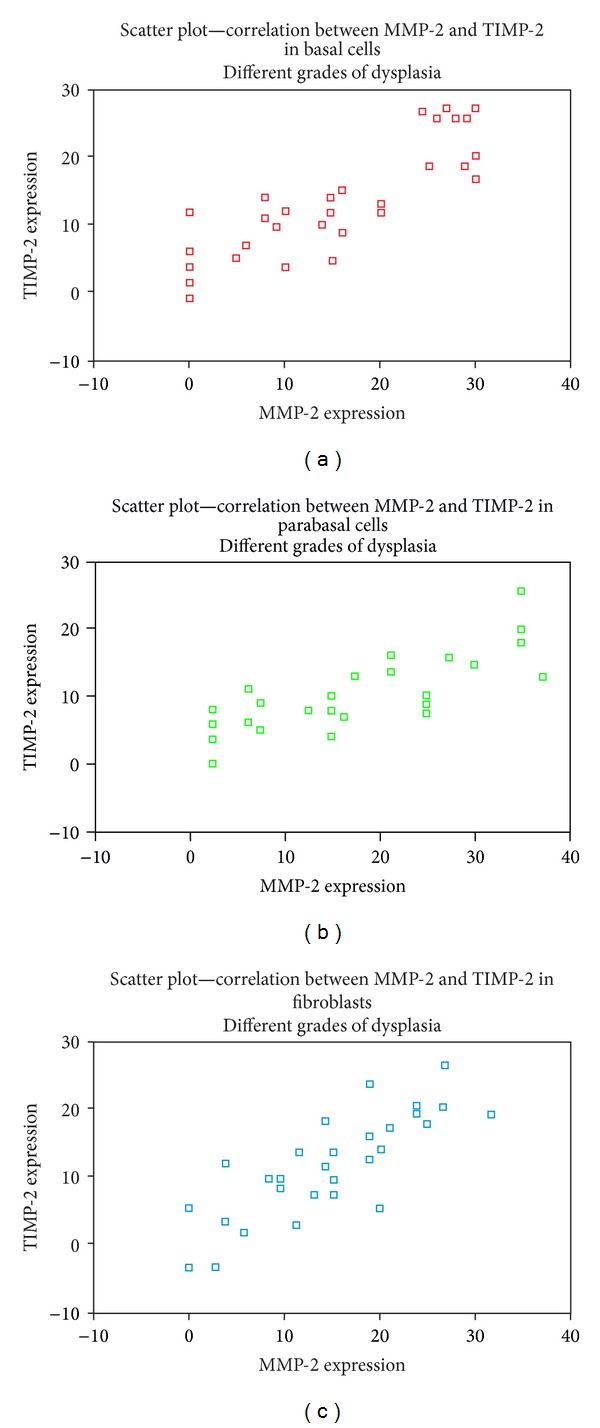
Scatter plot showing the correlation between MMP-2 and TIMP-2 in the cells observed through different grades of dysplasia. (a) Basal cells; (b) parabasal cells; (c) fibroblasts.

The cellular expression of MMP-2 and TIMP-2 through different grades of dysplasia by Spearman's correlation (Figures 4(a), 4(b), and 4(c)) showed a positive correlation for all the cells studied for MMP-2 and TIMP-2 (rho = 0.890–0.895) in all the grades of dysplasia. The same was also true for MMP-2 and TIMP-2 (rho = 0.666; 0.705 and 0.747) in mild, moderate, and severe dysplasia and *P* < 0.001.

## 4. Discussion

The pathogenesis of oral cancer is a multistep process which involves sequential progression of normal mucosa through a continuous spectrum of lesions, including hyperplasia, dysplasia, carcinoma in situ, and invasive carcinoma [14, 15]. In this study we sought to determine the expression and the correlation of MMP-2 and TIMP-2 in oral leukoplakia through different grades of oral epithelial dysplasia as these proteases appear to be important in the early phases of cancer progression including local invasion and micrometastasis [16]. Under pathological conditions, the changes in TIMP levels are considered to be important because they directly affect the levels of MMP activity.

The expression of MMP-2 and TIMP-2 in the basal and parabasal cells of the overlying epithelium as well as in the fibroblasts of the lamina propria in most of the cases of dysplasia has been shown by other investigators [16, 17]. This expression of MMP-2 in both the epithelium and the connective tissue components in a dysplastically altered tissue suggests a synergism between the two enzymes.

The MMP-2 mRNA was originally believed to be derived from the epithelial cells based on the observation that MMP-2, secreted in the latent proform, was activated by the loss of 80 amino acids from its N terminal residue of type IV procollagenase [18]. The current understanding is that the tumor cells have docking sites for binding the MMPs secreted by stromal cells, thus highlighting the epithelial mesenchymal interactions. Two mechanisms have been put forth to explain the expression of MMP-2 in the dysplastically altered epithelial cell adjacent to an intact basement membrane by the fibroblast. It is postulated that fibroblast derived MMP-2 is activated by a factor secreted from the cancer cell called EMMPRIN, an extracellular matrix metalloproteinase inducer. After being secreted by the fibroblast, pro-MMP-2 may bind to the cancer cells possibly to the MT-MMPs situated on the surface of their plasma membrane [19]. Furthermore, activated MMP-2 binds to the cell surface of neoplastically altered cells and endothelial cells via *α*v*β*3 integrin. Thus, it has been hypothesized that the secretion of MMP-2 may be by stromal cells, as well as by neoplastically altered epithelial cells. Both the epithelial and the stromal cells being the source of MMP-2 and TIMP-2 in ECM degradation and subsequent cellular motility may facilitate tumor invasion and metastasis [20].

The absence of expression of MMP-2 and TIMP-2 under normal condition is perhaps due to its secretion in minimal amount in the normal tissue [21], suggesting a reduced availability of pro-MMP-2, which is critical for the activation of MMP-2 [22]. However, as the endothelial cells lining the blood vessels were positive for both MMP-2 and TIMP-2 in normal mucosa, it has been attributable to secretion of relatively high concentrations of immunoreactive, but functionally inactive metalloproteinases by the endothelial cells [23]. The absence of demonstrable proteolytic activity by the endothelial cells was owing to production of large amounts of TIMP-1 and -2 by the endothelial cells which forms a complex with and inhibits MMP activity [24]. Thus our findings in normal mucosa suggest a balanced production of MMP-2 and its inhibitors in order to maintain a normal physiologic condition.

Our observation of negative-to-weak expression of MMP-2 in cases of mild dysplasia, weak-to-moderately positive expression in moderate and severe dysplasia in the basal and parabasal cells of the epithelium and fibroblasts of lamina propria is similar to those made by and Sarioğlu et al. [17] and Määttä et al. [10], where an increase in the expression of MMP-2 was noted in preneoplastic lesions of larynx and vulvar epithelium, respectively. These observations suggest that the neoplastically altered cells have heterogeneous potential to produce MMP-2. Since the production of MMP-2 in normal cells is regulated by the growth factors and cytokines, this heterogeneity seems to be due to differential properties of individually altered neoplastic cells in terms of the expression of specific receptors for these agents [25].

Our observation of increased MMP-2 positivity with decreasing architectural organization and increased atypia may be related to the regulation of MMP-2 at the transcriptional and posttranscriptional levels. The regulation of MMP-2 involves several overlapping mechanisms as has been demonstrated in a study by Qin et al. [26], where it has been shown that the binding of MMP-2 promoter by transcription factors Sp1, Sp3, and AP-2 leads to constitutive expression of these proteases in astroglioma cells. In another study carried out by Ruiz et al. [27], the loss of AP-2*α*, very early in the progression of cancer before it is clinically significant, suggests that the loss of AP-2*α* may potentially influence cancer progression by deregulating MMP-2 gene.

The other possible mechanism that modulates the transcriptional activity of MMP-2 gene is due to the Ras mutation, which is found in approximately 50% of all oral cancer patients in South Asian population [28], majority of which are thought to be associated with the habit of chewing tobacco. K-Ras gene products, c-K (B)-Ras, are believed to modulate the expression of MMP-2 expression by growth factors through the Ras/MAPK and AKT pathways. Activation of oncogenic K-Ras in oral cavity may thus represent early stages of tumor progression suggesting a possible inducing effect of the Ras oncogene on metastasis by activation of MMP-2/type IV collagenase [29].

The expression of TIMP-2 in our observation varied from weak to moderately positive corresponding with the grade of dysplasia. The proportion of cells expressing TIMP-2 was higher in cases of severe dysplasia as compared to those of mild dysplasia and normal buccal mucosa. Also a positive correlation was established between MMP-2 and TIMP-2 expressions in different cells observed as well as different grades of dysplasia. These findings are in agreement with Määttä et al. [10] that showed increased expression of TIMP-2 with increase in dysplastic features in the vulvar intraepithelial neoplasia (VIN I–III).

TIMP-2 is believed to suppress tumor invasion by inhibiting MMPs. However, TIMP-2 also has an important role in the activation mechanism of MMP-2 forming a pro-MMP-2/TIMP-2/MT1-MMP ternary complex on the cell surface, promoting hydrolysis of pro-MMP-2 to its active-form MMP-2 and resulting in degradation of ECM. The expression of TIMP-2 thus correlates with the metastatic ability and poor prognosis [30]. Hence contrary to the previously documented antitumor effects of TIMPs; TIMP-2 immunoexpression could have a tumor-promoting role in tumor recurrence and poor prognosis.

Overexpression of both MMP-2 and TIMP-2 at varying levels demonstrates the dual role of TIMP-2 in regulating MMP-2 processing and invasion. Recent studies have suggested that the regulation of MMP-2 by TIMP-2 is in a dose-dependent manner [31]. Although previous studies have indicated that TIMP-2 can inhibit MMP-2 activation due to its ability to bind and inhibit MMP-2, endogenous physiological upregulation of TIMP-2 expression can promote MMP-2 activation and subsequent cell invasion. According to this model, a half molar ratio of TIMP-2 to MT1-MMP is said to be theoretically optimal for MMP-2 activation. A study carried out in the invasive glioblastoma cells showed that increase in local TIMP-2 concentrations has the effect of initially stimulating MMP-2 activation, whereupon exceeding amounts of TIMP-2 are inhibitory to MMP-2 activation [32]. Thus, on one hand TIMP-2 inhibits MMP-2 activity while on the other hand it is necessary for MMP-2 activation as a major cofactor [33].

## 5. Conclusion

In our study, an increase in the expression of both MMP-2 and TIMP-2 from normal oral epithelium to severe dysplasia suggested that the activation of MMP-2 is dependent on TIMP-2 acting as a cofactor, thereby providing a microenvironment that enhances the spread of tumor cells. Monitoring of MMP activity in the dysplastic epithelium could be a pertinent approach in patients with potentially malignant oral disorder and thereby determining the patients at true risk of developing malignancy. However, our preliminary observation of increase in the expression of MMP-2 and TIMP-2 and their positive correlation through different grades of oral dysplasia needs to be further validated. Since the proportions of cells positive by immunohistochemistry does not always reflect the activity of the expressed protein as the expression of MMP-2 and TIMP-2 is regulated at multiple steps, including transcription, translation, and posttranslational modifications, use of advanced techniques like ELISA, Gelatin zymography, and RT-PCR could further validate our findings.

## Figures and Tables

**Table 1:** The clinical and histological details of patients included in the study.

No.	Age	Sex	Habit	Site	Clinical diagnosis	Histological grade
1	46	M	Tobacco smoking	Buccal mucosa	Leukoplakia	Mild dysplasia
2	55	M	Betel quid + tobacco	Buccal mucosa	Leukoplakia	Mild dysplasia
3	55	M	Betel quid + tobacco	Alveolar mucosa	Leukoplakia	Mild dysplasia
4	53	M	Tobacco smoking	Buccal mucosa	Leukoplakia	Mild dysplasia
5	41	M	Tobacco smoking	Buccal mucosa	Leukoplakia	Mild dysplasia
6	61	M	Tobacco smoking	Buccal mucosa	Leukoplakia	Mild dysplasia
7	43	M	Betel quid + tobacco	Alveolar mucosa	Leukoplakia	Mild dysplasia
8	40	M	Tobacco smoking and betel quid	Buccal mucosa	Leukoplakia	Mild dysplasia
9	60	M	Betel quid + tobacco	Buccal mucosa	Leukoplakia	Mild dysplasia
10	52	M	Tobacco smoking	Buccal mucosa	Leukoplakia	Mild dysplasia
11	65	M	Tobacco smoking and betel quid	Buccal mucosa	Leukoplakia	Moderate dysplasia
12	57	M	Tobacco smoking	Buccal mucosa	Leukoplakia	Moderate dysplasia
13	47	M	Tobacco smoking and betel quid	Buccal mucosa	Leukoplakia	Moderate dysplasia
14	61	M	Tobacco smoking and betel quid	Buccal mucosa	Leukoplakia	Moderate dysplasia
15	42	M	Tobacco smoking	Alveolar mucosa	Leukoplakia	Moderate dysplasia
16	62	M	Tobacco smoking	Buccal mucosa	Leukoplakia	Moderate dysplasia
17	65	M	Tobacco smoking and betel quid	Alveolar mucosa	Leukoplakia	Moderate dysplasia
18	63	M	Tobacco smoking	Buccal mucosa	Leukoplakia	Moderate dysplasia
19	53	M	Tobacco smoking	Buccal mucosa	Leukoplakia	Moderate dysplasia
20	62	M	Betel quid + tobacco	Buccal mucosa	Leukoplakia	Moderate dysplasia
21	60	M	Tobacco smoking and betel quid	Buccal mucosa	Leukoplakia	Severe dysplasia
22	62	M	Betel quid + tobacco	Buccal mucosa	Leukoplakia	Severe dysplasia
23	57	M	Tobacco smoking and betel quid	Buccal mucosa	Leukoplakia	Severe dysplasia
24	65	M	Betel quid + tobacco	Buccal mucosa	Leukoplakia	Severe dysplasia
25	45	M	Betel quid + tobacco	Buccal mucosa	Leukoplakia	Severe dysplasia
26	51	M	Tobacco smoking and betel quid	Buccal mucosa	Leukoplakia	Severe dysplasia
27	51	M	Tobacco smoking and betel quid	Buccal mucosa	Leukoplakia	Severe dysplasia
28	65	M	Betel quid + tobacco	Buccal mucosa	Leukoplakia	Severe dysplasia
29	67	M	Tobacco smoking and betel quid	Buccal mucosa	Leukoplakia	Severe dysplasia
30	64	M	Betel quid + tobacco	Buccal mucosa	Leukoplakia	Severe dysplasia

**Table 2:** Expression of MMP-2 and TIMP-2 in cells of basal and parabasal layers and fibroblasts in all the grades of dysplasia.

Cells	Dysplasia	MMP-2 score			Total	Chi-square	*P* value*	TIMP-2 score			Total	Chi-square	*P* value*
		−	+	++				−	+	++			
Basal	Normal	10	0	0	10	35.833	<0.001	10	0	0	10	39.461	<0.001
	Mild	5	5	0	10			1	9	0	10		
	Moderate	0	7	3	10			0	8	2	10		
	Severe	0	4	6	10			0	7	3	10		
	Total	**15**	**16**	**9**	**40**			**11**	**24**	**5**	**40**		

Parabasal	Normal	10	0	0	10	33.461	<0.001	10	0	0	10	32.923	<0.001
	Mild	5	5	0	10			3	7	0	10		
	Moderate	1	9	0	10			0	10	0	10		
	Count	1	5	4	10			0	9	1	10		
	Total	**17**	**19**	**4**	**40**			**13**	**26**	**1**	**40**		

Fibroblast	Normal	10	0	0	10	46.582	<0.001	10	0	0	10	38.156	<0.001
	Mild	1	9	0	10			1	9	0	10		
	Moderate	0	9	1	10			0	10	0	10		
	Severe	0	5	5	10			0	9	1	10		
	Total	**11**	**23**	**6**	**40**			**11**	**28**	**1**	**40**		

MMP-2: matrix metalloproteinase-2; TIMP-2: tissue inhibitor of metalloproteinase-2.

*Kruskal-Wallis test.

**Table 3:** The mean rank of MMP-2 and TIMP-2 in different grades of dysplasia.

Cells	Markers	Dysplasia	Cases	Mean rank	Chi-square	*P* value*
Basal cells	MMP-2	Normal	10	8.00	27.462	<0.001
		Mild	10	15.40		
		Moderate	10	26.30		
		Severe	10	32.30		
	TIMP-2	Normal	10	6.00	26.125	<0.001
		Mild	10	19.15		
		Moderate	10	26.05		
		Severe	10	30.80		

Parabasal cells	MMP-2	Normal	10	9.00	22.065	<0.001
		Mild	10	17.10		
		Moderate	10	24.55		
		Severe	10	31.35		
	TIMP-2	Normal	10	7.00	25.495	<0.001
		Mild	10	17.50		
		Moderate	10	26.70		
		Severe	10	30.80		

Fibroblasts	MMP-2	Normal	10	6.00	26.599	<0.001
		Mild	10	19.25		
		Moderate	10	25.25		
		Severe	10	31.50		
	TIMP-2	Normal	10	6.00	26.660	<0.001
		Mild	10	19.10		
		Moderate	10	25.50		
		Severe	10	31.40		

MMP-2: matrix metalloproteinase-2; TIMP-2: tissue inhibitor of metalloproteinase-2.

*Kruskal-Wallis test.
